# Time-domain THz spectroscopy of the characteristics of hydroxyapatite provides a signature of heating in bone tissue

**DOI:** 10.1371/journal.pone.0201745

**Published:** 2018-08-23

**Authors:** Marie Plazanet, Jordanka Tasseva, Paolo Bartolini, Andrea Taschin, Renato Torre, Christèle Combes, Christian Rey, Alessandro Di Michele, Mariana Verezhak, Aurelien Gourrier

**Affiliations:** 1 Univ. Grenoble Alpes, CNRS, LIPhy, Grenoble, France; 2 Dipartimento di Fisica e Geologia, Università degli Studi di Perugia, Perugia, Italy; 3 European Laboratory for Non-Linear Spectroscopy (LENS) and Dip. di Fisica ed Astronomia, Università di Firenze, Sesto Fiorentino, Italy; 4 CIRIMAT, Université de Toulouse, CNRS, INPT-ENSIACET, Toulouse, France; Institute of Materials Science, GERMANY

## Abstract

Because of the importance of bone in the biomedical, forensic and archaeological contexts, new investigation techniques are constantly required to better characterize bone ultrastructure. In the present paper, we provide an extended investigation of the vibrational features of bone tissue in the 0.1-3 THz frequency range by time-domain THz spectroscopy. Their assignment is supported by a combination of X-ray diffraction and DFT-normal modes calculations. We investigate the effect of heating on bone tissue and synthetic calcium-phosphates compounds with close structure and composition to bone mineral, including stoichiometric and non-stoichiometric hydroxyapatite (HA), tricalcium phosphate, calcium pyrophosphate and tetracalcium phosphate. We thus demonstrate that the narrow vibrational mode at 2.1 THz in bone samples exposed to thermal treatment above 750 °C arises from a lattice mode of stoichiometric HA. This feature is also observed in the other synthetic compounds, although weaker or broader, but is completely smeared out in the non-stoichiometric HA, close to natural bone mineral composition, or in synthetic poorly crystalline HA powder. The THz spectral range therefore provides a clear signature of the crystalline state of the investigated bone tissue and could, therefore be used to monitor or identify structural transitions occurring in bone upon heating.

## Introduction

Bone grafts are essential in dental and bone clinical practice to bridge pathological fractures, fill large defects or strengthen a diseased tissue. However, due to numerous problems associated with donor site morbidity and limited availability (autologous grafts) or with immunological response and infectious risk (allografts, xenografts), synthetic bone substitutes have become recognized as a valuable alternative [1]. A wide variety of calcium-phosphate based ceramic scaffolds have thus been developed and combined with growth factors and stem cells to improve the osteoconductive and osteoinductive properties [2]. Those mostly consist of hydroxyapatite (HA) and/or *β*-tricalcium phosphate (*β*-TCP) manufactured in the form of pastes, granules, coatings or bulk materials [3]. Animal bone, considered as a waste product from the food industry, has extensively been tested as a natural resource for HA production by high temperature heating (> 1100 °C). Bovine bone, for example, is commercially used to fabricate PepGen P-15^®^, Endobon^®^ and Cerabone^®^ biomaterials [3] and there is a high potential for the use of other types of bones (fish, ovine, porcine…). Independently of the nature of the starting materials, the precise chemistry and morphometry of those end-products have been shown to influence the tissue formation through the activity of bone cells [4, 5] and therefore require a precise control during (or at least following) the manufacturing process.

Since atomic or molecular scale sensitive techniques are required to monitor the structural evolution of bone mineral or synthetic HA, vibrational spectroscopy is widely used to characterize the effect of heating on bone and calcium phosphate minerals, often in conjunction with X-ray diffraction (XRD), scanning electron microscopy (SEM) and thermogravimetric analysis [6–12]. Interestingly, similar studies were also reported in the fields of forensic science and archeology [13–16]. Although undertaken in very different contexts, those studies provide very complementary mechanistic information. The infra-red (IR) and Raman spectroscopic investigations were based on well established assignment of the vibrational bands from 400 up to 3600 cm^−1^ [17–19], or even down to ∼100 cm^−1^ [20]. Both collagen and mineral phases present characteristic peaks in this spectral range.

Those studies provided a fine characterization of bone transformations upon heating. It was shown indeed that, up to 300 °C, the mineral crystalline phase is only slightly affected by the loss of hydrogen phosphate and carbonates ions, as well as by a moderate crystal growth [21–25]. In this temperature range, the most important changes concern the collagen degradation from denaturation to calcination above 300 °C, leading to a brown and black coloration of the samples. Beyond 600 °C, the color fades and turns white around 800 °C indicating a transformation into a pure mineral phase. The loss of organic matter is accompanied by important crystallographic changes in the mineral phase starting around 750 °C, which were accurately characterized by X-rays and TEM investigations [24, 26, 27]. Beyond this characteristic temperature, the crystallite average dimension then increases from tens to thousands of nm in conjunction with sintering processes [12]. This structural transition is critical for the mechanical, microstructural and surface properties of the material [28, 29] and its precise identification would therefore provide a very important cue of the transformation process induced by heating.

One drawback of these studies is that they are generally performed on complex instruments or involve specific sample preparation, which is relatively impractical for fast and cost-effective screening of a large number of samples. Hence, a method that could provide a vibrational signature of this transition would prove highly beneficial. Up to now, no striking features were revealed in the low frequency (far-IR) range which can be advantageously investigated by THz time-domain spectroscopy (THz-TDS). This technique was mostly employed for imaging purposes [[30, 31]]. So far, THz-TDS measurements mainly showed a continuous increase of the absorption coefficient as a function of frequency [32–34] and did not reveal any underdamped (absorption) feature in bones or HA. However, the characterization of phonon lattice modes which are expected in the THz frequency range, could provide valuable information since they are particularly sensitive to crystalline environment.

Because THz-TDS can easily be implemented at relatively low cost using commercial instruments, we undertook an investigation of heated cortical bone of a bovine femur heated up to 1000 °C, covering the frequency range from 0.1 to 3 THz. An additional set of synthetic calcium phosphate powders, of direct or related interests for bone-like materials were also studied: stoichiometric HA, *α* and *β*-tricalcium phosphate, *β*-calcium pyrophosphate and tetracalcium phosphate. To complete the work, we also characterized a non-stoichiometric commercial HA as received and heated above 800°C. We observed the appearance of a well defined peak at ∼ 2.1 THz in the absorption spectra of bone above 750 °C, simultaneous to the well characterized recrystallisation of the mineral part. We then combined the spectroscopic measurements with X-ray diffraction, enabling structural characterization, and density functional theory (DFT) simulations of the crystal phonon modes of the bone mineral components that are responsible for the absorption spectra. As demonstrated in the following sections, we could identify the signature of the crystalline state of bone tissue from the THz phonon spectrum.

## Materials and methods

### Bone samples

**Fig 1:**
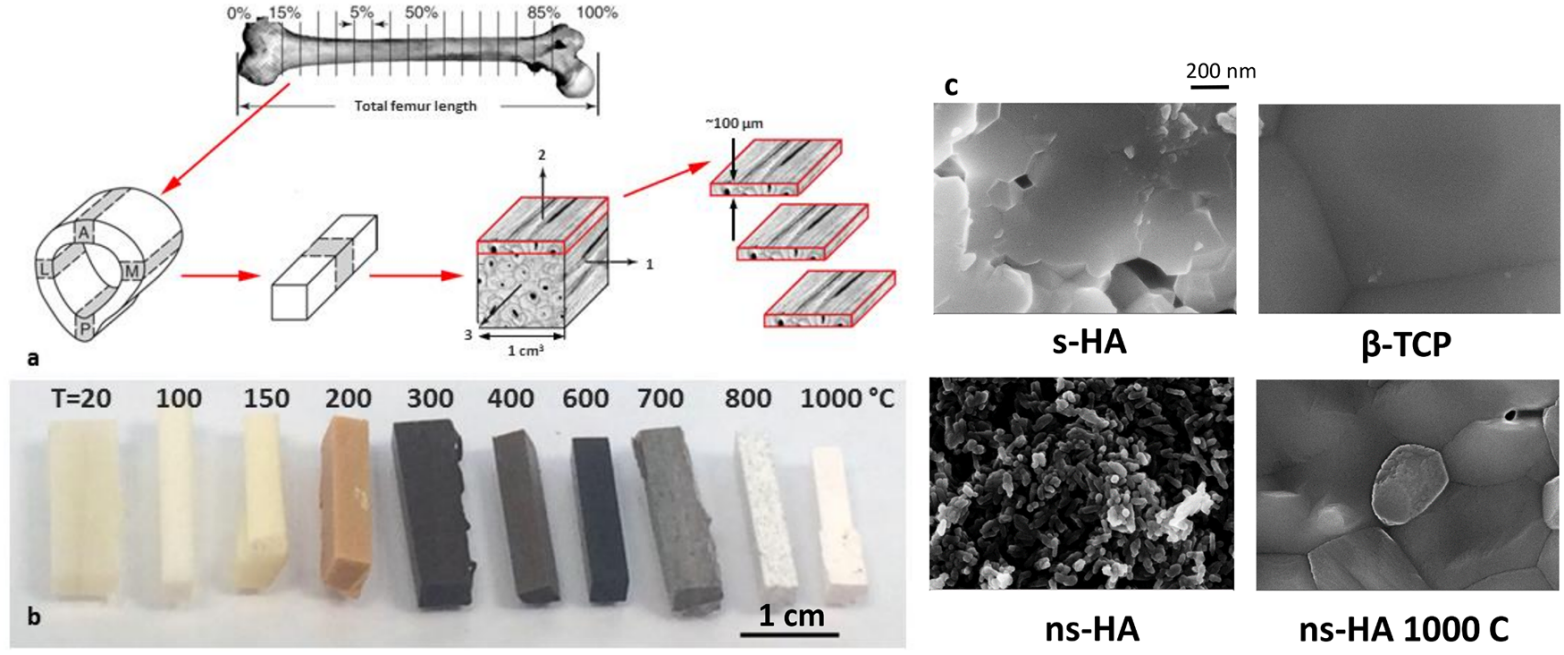
Macroscopic and nanoscale evolution of HA upon heating. a) sample preparation scheme; b) color changes of bone sticks as a function of temperature; scale bar 10 mm; c) SEM images showing the nanoscale heating effect on ns-HA, and compared to s-HA and *β*-TCP. Scale bar is 200 nm for all the images.

The samples were prepared from a bovine femur obtained from the local slaughterhouse, 38120 Fontanil-Cornillon, France. The periosteum and marrow were mechanically removed and a transverse cross-section of ∼15 mm in thickness was sawed in the diaphysis, fixed with ethanol 70% v/v, dehydrated in a graded series of ethanol solutions of 80% v/v, 90% v/v and 100% v/v. A cortical block of 10x10x10 mm^3^ was selected in the medial quadrant. Light microscopy examination revealed a homogeneous fibro-lamellar structure with alternating layers of fibrous and lamellar bone with characteristic dimensions of 100 *μ*m. Longitudinal sections of approximately 100 to 150 *μ*m in thickness were cut from this block using a high precision low-speed diamond saw (Mecatome T210, PRESI) (Fig 1a).

One section was used as a control, while the others were heated to 100, 200, 300, 400, 500, 600, 650 700, 750, 800, 850 and 1000 °C in an unvented oven (Carbolite Gero, ELF) and cooled in air. The samples were treated sequentially: each sample was inserted at the final heating temperature of the previous one, the next temperature was reached, followed by curing for 10 minutes. The temperature precision of the thermocouple was ∼ 2-3 °C and the sample reached the given temperature in less than a minute.

### Synthetic and commercial compounds

We investigated a series of calcium phosphate model compounds synthesized in CIRIMAT (Toulouse, France). These include: the *α* and *β* phases of tricalcium phosphate (*α*- and *β*-TCP, Ca_3_(PO_4_)_2_), *β* phase of calcium pyrophosphate (*β*-CPP, Ca_2_P_2_O_7_), tetracalcium phosphate (TTCP, Ca_4_(PO_4_)_2_O) and stoichiometric hydroxyapatite (s-HA, Ca_5_(PO_4_)_3_OH).

Briefly, *α*-TCP was prepared by heating *β*-TCP at 1350°C for 1 hour in a platinum crucible and quenching in liquid nitrogen. *β*-TCP was synthesized according to the method described by Heughebaert and Montel [35]. *β*-CPP was obtained by heating dicalcium phosphate dihydrate at 900°C for 3 hours [36]. TTCP was prepared by heating a thoroughly ground mixture of equimolar quantities of calcium carbonate and dicalcium phosphate dihydrate at 1400°C, in a platinum crucible, for 6 hours, and quenching in liquid nitrogen. The stoichiometric HA was synthesized by double decomposition of aqueous solutions of calcium nitrate and diammonium phosphate at boiling temperature; after filtration the precipitate was heated at 1000°C for 15 hours [37]. All samples were controlled by X-ray diffraction and FTIR spectroscopy. They were not found to contain any detectable foreign crystalline phases.

Eventually, we studied a powder of non-stoichiometric hydroxyapatite (refered as ns-HA) ref. 55497 from Sigma-Aldrich. This last powder was also heat treated in the same conditions applied to the bone slices, at temperatures of 650, 750, 800 and 1000°C.

### THz Time Domain Spectroscopy setup

For measuring the frequency dependence of the refractive index, *n*(*ω*), and the absorption coefficient, *α*(*ω*), of bone samples, we used a THz-TDS set-up in transmission configuration, which enabled us to study the optical parameters in the range of frequency 0.1-4 THz. The THz pulses are produced by a photoconductive antenna excited by a femtosecond optical laser pulse (λ = 780 nm, Δt = 120 fs at 100 MHz) and biased with a sinusoidal voltage for a lock-in detection. The emitted THz radiation is collimated by an off-axis parabolic mirror then focused on the sample by a second parabolic mirror. By other two off-axis parabolic mirrors, the THz beam is again collimated and finally focused on a second photoconductive antenna for its detection. This second antenna works as current generator: free electron-couple carriers are produced into the antenna gap by a second optical laser pulse whilst the THz field now acts as a bias. Thus, the temporal evolution of the photocurrent amplitude, acquired by the changing time delay between excitation and gate pulses, is directly related to the electric field amplitude of the THz radiation. This current signal is amplified by a lock-in amplifier and digitalized by an acquisition board. By a homemade software we simultaneously acquire the processed signal and delay line encoder, and retrace the final time-dependent THz field. The whole THz set-up is enclosed in a nitrogen purged chamber for removing the water vapor contribution present at the THz frequencies spanned by the experiment. Exhaustive description about our THz-TDS set-up can be found in previously published papers [38–40].

The ratio between the THz field transmitted after the sample, *E*_*t*_(*ω*), and the incident field, *E*_*i*_(*ω*), is named transfer function of the material, *H*(*ω*) [38]. The optical properties of the material, *n*(*ω*) and *α*(*ω*), can be fully extracted by the experimental transfer function, *H*_*exp*_(*ω*), which can be obtained by the ratio of the complex Fourier transform of the sample and the reference signal. However, the analytical expression for *H*(*ω*) is not given in a closed-form and the optical parameters, *n*(*ω*) and *α*(*ω*), can not be calculated without an iterative algorithm. The complexity of this process strongly depends on the nature of the THz signal and the sample thickness. Recently, we have implemented an innovative experimental procedure and numerical method to analyze the transmission THz-TDS signal of samples composed of multiple thin layers [39, 40]. The numerical method is quite complex and is based on a polynomial fit of the optical parameters. By this procedure, we can safely extract the real frequency dependence of the transmission parameters, totally removing the multiple reflections due to the Fabry-Perot effect. This data analysis enables the measurement of the *n*(*ω*) and *α*(*ω*) in an absolute scale, obtaining a complete quantitative set of the THz transmission parameters for bone samples.

### X-ray diffraction

The X-ray diffraction data were recorded on a laboratory diffractometer at the Physics and Geology department of the University of Perugia, using the radiation of the Cu K_*α*_ (1.5418 Å). A monochromatic incoming beam was obtained using a pyrolytic graphite monochromator which allows removing the K_*β*_ line. Higher order wavelength contamination was avoided using a scintillation detector having adequate energy resolution. The bone sections were mounted on a holed aluminium plate.

### Scanning electron microscopy

The SEM characterization was performed on FE-SEM LEO1525 Zeiss. Powder samples were glued on a graphite layer and metalized by depositing a Chromium layer of 8 nm thickness.

### DFT calculations

The phonon density of states was calculated using density functional therory (DFT) implemented in the VASP 5.4 software package. For each compound, the crystalline structure from experimental diffraction data (HA [41], *β*-TCP [42], *β*-CPP [43], TTCP [44]) was used as the initial configuration. Atomic positions were optimized without any change of cell volume or shape in order to find the minimum energy structure using PAW-PBE ultrasoft pseudopotentials and an energy cut-off of 700 eV for the plane wave basis. The optimized structures are in good agreement with the experimental ones, with averaged discrepancies in atomic distances smaller than 1 %. The normal modes calculation was then performed using the linear response theory (IBRION = 7), and the IR absorption intensities were calculated according to the methods proposed by Karhanek et al. [45]. In all the calculations, the three translational modes appear at negative frequencies, although not lower than -0.1 THz. All the other frequencies were positive, except for *β*-TCP where two additional modes have frequencies between 0 and -1.0 THz. The calculated frequencies are in good agreement with the calculations of reference [46]. The spectral characteristics were convolved with a FWHM 0.1 THz Gaussian to account for instrumental resolution function. As a second test, we computed the full IR spectrum of HA and *β*-TCP, and got results in good agreement with experimental data in the 400—1400 cm^−1^ frequency range, as published in references [14] and [47] respectively.

## Results

### SEM

As previously reported [21], no significant color change was observed until 150-200 °C where the bone blocks progressively became brown (200 °C), black (300-600 °C) before turning gray (600-700 °C) and white (> 800 °C). Note the gray speckles at 800 °C which tend to indicate a heterogenous transformation (See Fig 1b). At the nanoscale, the ns-HA which is close to bone mineral structure and composition is formed of ∼ 20 nm diameter nanocrystals which fuse and grow up to hundreds of nanometers above 750 °C (See Fig 1c).

### THz-TDS

**Fig 2:**
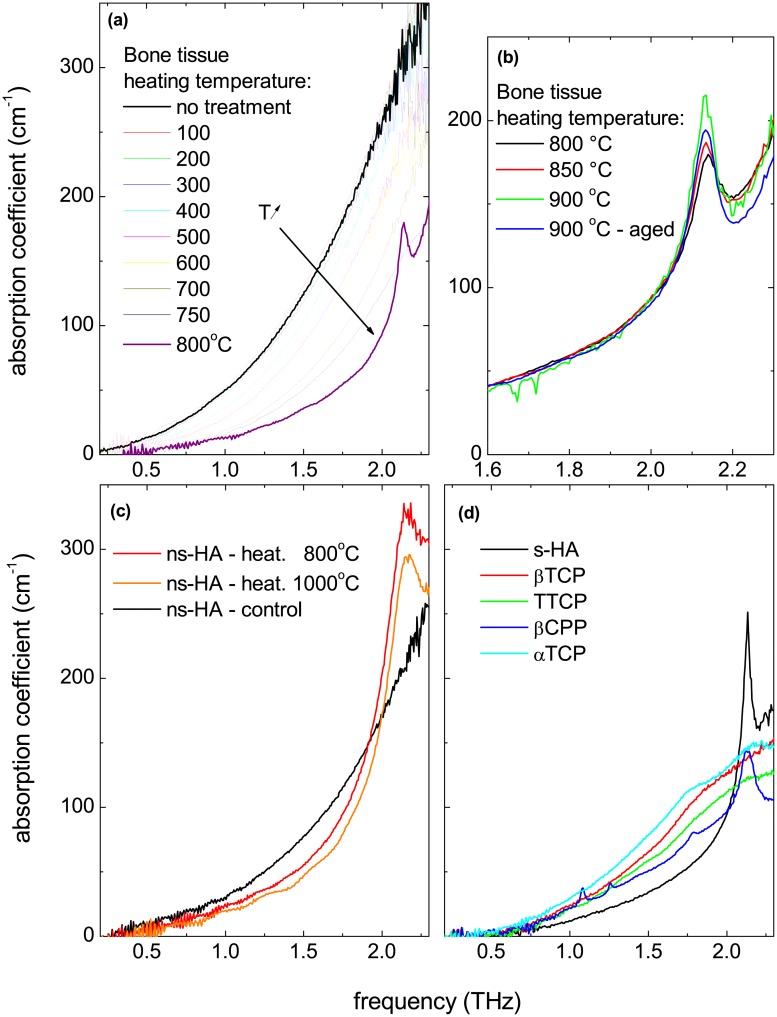
THz absorption spectra of bone and synthetic reference calcium phosphate compounds. *a*) absorption coefficients of an untreated bone sample and a series of temperature-treated samples up to 800 °C; *b*) absorption coefficients from a series of high-temperature (from 800 to 900 °C) treated bone samples; *c*) absorption coefficients of commercial ns-HA powder samples, untreated and treated at different temperatures; *d*) absorption coefficients from a series of reference calcium phosphate compounds: stoichiometric hydroxyapatite s-HA, TTCP, *α* and *β*-TCP, and *β*-CPP.

The absorption spectra measured by THz-TDS on control and samples thermally treated within a temperatures range of 100–800 °C are presented in Fig 2a. The overall decrease of the absorption coefficient with temperature can be assigned to the progressive loss of collagen content in the bone samples. The spectra measured on the samples treated at temperatures ≥800°C show a new absorption peak at about 2.1 THz (see Fig 2b), which intensity slightly increases up to 900 °C. After storing the sample for two weeks at room temperature, the spectrum slightly evolved, suggesting a slow relaxation of the bone structure in the samples (sample labeled as “aged” in Fig 2b).

In Fig 2c, we report the THz spectra of commercial HA powders, as received and treated at two different temperatures. Similarly to the bone samples, the ns-HA exhibits the 2.1 THz peak after heating at 800 °C and higher. We can therefore rule out the hypothesis that this peak is hidden in the samples heated at temperature lower than 750°C by the absorption background produced by the presence of organic components. The peak growth can therefore only be assigned to an increase of the size of the crystallites during the recrystallization process at ∼750°C and/or to a simultaneous ordering of the crystalline structure.

Since it is known that phase transitions and decomposition can take place in hydroxyapatite at different temperatures [48], we also investigated several compounds that may be present in the heated bone: *α*- and *β*-TCP, *β*-CPP, TTCP and s-HA. The corresponding THz absorption spectra (reported in Fig 2d) clearly show that the 2.1 THz vibrational band is particularly intense and narrow in s-HA, and is also well defined in *β*-CPP. The spectra of *α*-, *β*-TCP and TTCP are not featureless but only exhibit broad bands.

### X-ray investigation of heated HA

**Fig 3:**
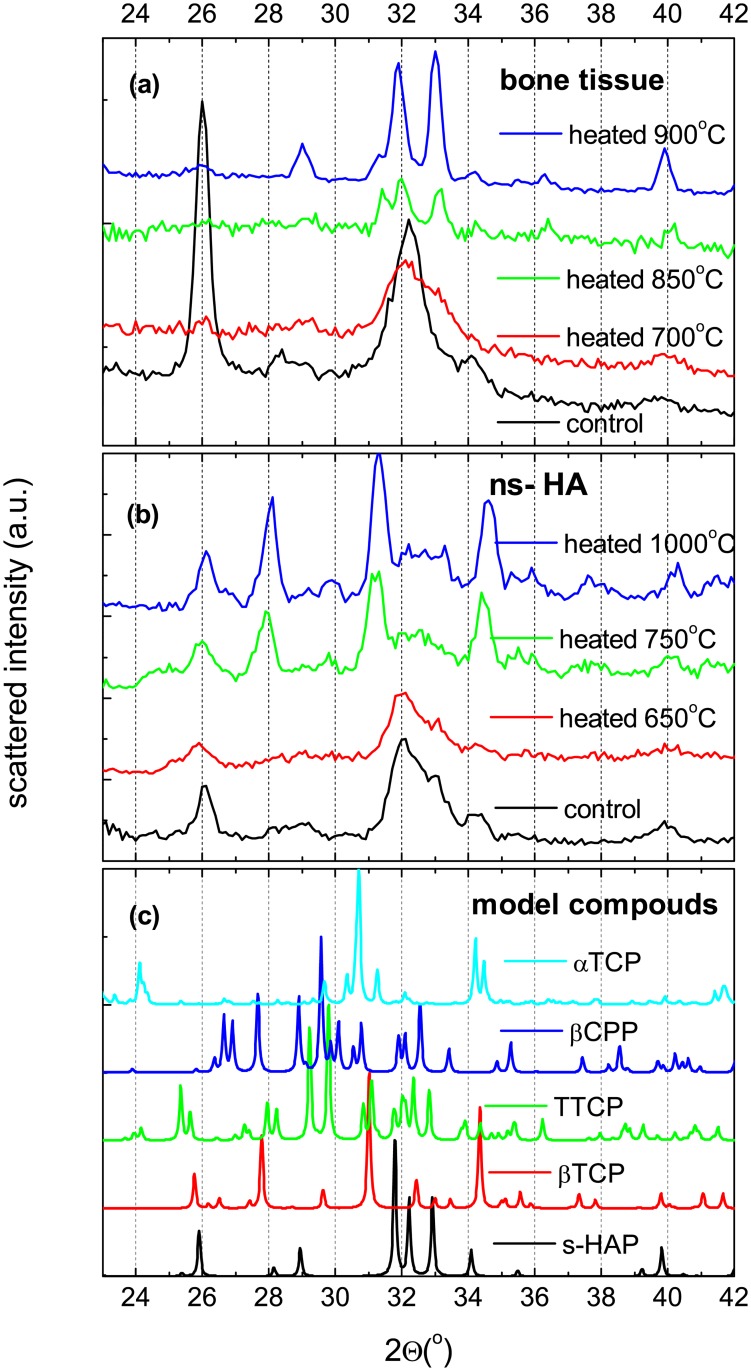
**X-ray diffraction patterns** collected from *a*) untreated and heat-treated bone tissues, and *b*) untreated and heat-treated commercial hydroxyapatite powder (ns-HA). *c*) Simulated patterns for synthetic calcium phosphate compounds based on refined structures [41–44].

X-ray diffraction was used to assess the existence of phase transitions previously reported in heat-treated samples. X-ray diffractograms, measured on bone samples and commercial hydroxyapatite (ns-HA) are presented in Fig 3a and 3b, respectively. Additionally, the simulated patterns from refined structures based on experimental data available from the literature (see Materials and methods section) are shown in Fig 3c. The comparison between experimental and calculated patterns has to be done keeping in mind the finite resolution of the diffractometer of about 0.5 °. According to the Scherrer equation that links the width of the diffraction peaks and the extension of the monocrystalline domains, our resolution corresponds to a characteristic size of a few nanometers, indicating that we are in any case limited by the instrument resolution and cannot evaluate the size of our crystallites.

Structural transitions clearly occur in bone tissue and ns-HA around 750 °C, although they do not result in the same crystalline phase. The ns-HA heated at 1000 °C exhibits Bragg peaks at 27.9, 31.0 and 34.5° that could be assigned to the presence of a significant quantity of *β*-TCP, overlapped with a HA ‘background’. Moreover, the broad feature around 38° can be assigned to the formation of CaO [49]. This observation is consistent with numerous studies on the thermal stability of non-stoichiometric HA, that are known to undergo various transformations upon heating [48, 50]. Indeed, the FTIR spectrum of the ns-HA before heating exhibits two bands at 870 and 1140 cm^−1^ that could be assigned to the presence of ${\text{HPO}}_{4}^{2-}$ ions, in agreement with the calcium deficiency of the apatite. Upon heating, the ns-HA decomposes in various compounds depending on its Ca/P ratio, in this case into TCP and HA. The FTIR spectrum of the heated ns-HA sample also reflects the TCP and HA decomposition (see S2 Fig). While in stoichiometric HA (s-HA), we only expect a phase transition from a monoclinic (space group *P*2_1_/*b*) to a hexagonal (*P*6_3_/*m*) lattice but no decomposition.

The phase transformation in heated bone tissue appears to be different from the one occurring in ns-HA. The X-rays diffractograms of the bone samples heated above 750 °C do not indicate the formation of *β*-TCP nor of CaO. The observed differences in diffraction pattern with the s-HA could simply be caused by the remaining substituting ions and, even after crystal growth, a less crystalline phase than what would be measured on perfect s-HA.

### DFT calculation of normal modes of vibration

**Fig 4:**
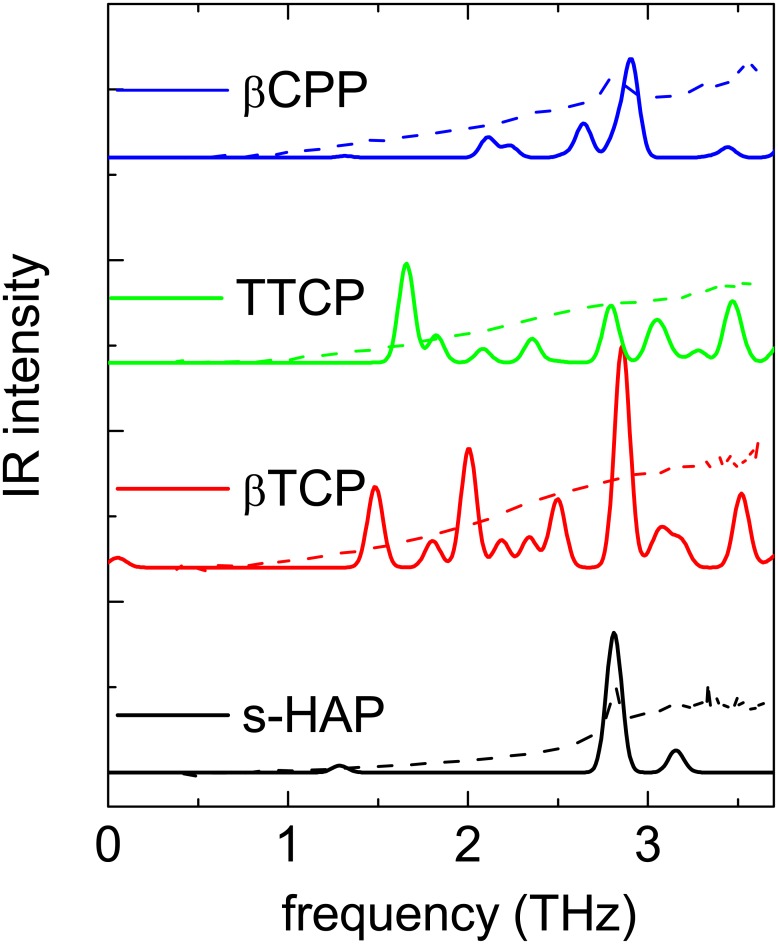
Simulation of THz spectra (continuous lines) by calculation of the normal modes of vibrational with DFT and experimental THz absorption data (dotted lines). In order to facilitate the visual comparison of these spectra, we adapted the experimental amplitude and frequency scales to match the 2.1 THz measured peak with the 2.8 THz calculated peak.

In Fig 4 we compare the DFT-calculated absorption spectra of some of the investigated compounds (solid lines) with the THz experimental data (dashed lines). The experimental data have been rescaled in frequency to match the peak positions of the simulated spectra (factor 1.33, so that 2.1 THz measured aligns with 2.8 calculated). It is important to note that the number of atoms per unit cell, N, is very different for each compound: N = 44, 60, 88 and 276 for HA, TTCP, *β*-CPP and *β*-TCP, respectively. 3N defines the total number of vibrational modes present in each compound, that are in all compounds approximately spread over the frequency range of 0-30 THz (without taking into account the isolated O-H stretching mode). We therefore observe, as expected, that the number of modes varies greatly between the considered compounds in the investigated frequency range.

We only find a qualitative agreement between the calculated and measured spectra. However, some interesting observations can be made. All compounds present vibrational modes in the 0.1-3.0 THz range, arising from lattice distortions. Among the compounds, the HA presents the most intense optical phonon at 2.8 THz, that we readily assign to the one observed in the experimental spectrum. The presence of an isolated single mode comes from the HA lattice structure: it has a small asymmetric unit cell enabling a low inhomogeneous broadening. This is also the case for the *β*-CPP compound which has a limited number of modes in this spectral region. The other compounds, TTCP and *β*-TCP, have more vibrational modes as can be expected from the number of atoms per unit cell. Moreover, in real samples, the modes are broadened by crystalline defects, so the overlapping of these broad peaks should lead to the observed continuum.

## Discussion

Our THz investigation of bone tissue clearly shows the appearance and increase of a well defined absorption peak at 2.1 THz induced by the thermal treatment, which is a characteristic lattice mode of HA crystals. This spectral change arises from the modifications in the mineral phase of bone tissue, induced in our study by the heating process. Indeed, in the low temperature range (≤ 750 °C) the organic components are gradually destroyed and the mineral phase present in bone tissue is composed mainly of nanometric crystals that do not contribute to any specific signature in the THz spectrum. The THz absorption spectrum therefore follows a monotonic slope that continuously lowers. In the high temperature range (≥ 800 °C) instead, only the mineral components are left in bone tissue and the THz measurements exhibit a well defined phonon vibration of 2.1 THz frequency that can be assigned to HA crystals. The central frequency and the width of the absorption peak prove that the HA crystals must be characterized by a well defined lattice structure (i.e. extended lattice, not affected by numerous impurities/defects). The X-ray diffraction investigations confirm that the thermally treated bone has a lattice structure similar to HA crystals. This scenario is in full agreement with other experimental results [24, 51].

**Fig 5:**
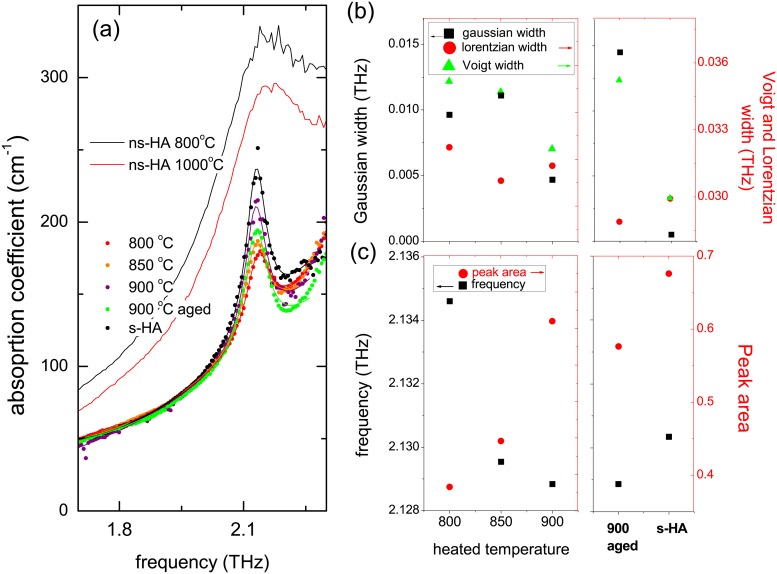
(a) Magnification of THZ-TDS spectra of bones and HA samples around the peak at 2.1 THz (dots) and fit with a Voigt peak profile on top of the background (lines). For ns-HA, only the experimental data are shown as lines; (b) evolution of the gaussian and lorentzian contributions to the Voigt profile for the heated bone samples (left), as well as aged sample and s-HA (right, as indicated along the *x*-axis.); (c) evolution of the peak frequency (left axis) and Voigt width (right axis).

A deeper analysis was carried out by fitting the spectra with a polynomial background and a Voigt function to extract the characteristics of the peak, i.e. frequency, shape and width. Their evolution as a function of temperature is plotted in Fig 5. From the observation of the spectra in Fig 5a, we note that the absorption coefficient in the 1.5-2.0 THz region, before the peak, is identical (within spectrum reproducibility) for bones samples heated at temperatures greater than 800°C and s-HA. This is in agreement with the view that bone HA becomes more stoichiometric after the heating transition at ∼750 °C. The non-stoichiometric heated powders indeed show a higher absorption coefficient, witnessing of a different composition, as for example mixed HA and TCP phases. Then, the fitting parameters of the peak give quantitative informations. The clearest behavior is shown by the peak area (Fig 5c), that increases with the heating temperature, up to the highest value in s-HA. The frequency, also plotted in Fig 5c, is another indicator of the crystalline state, since it shifts toward lowest values upon increasing the heating temperature. It even surprisingly reaches a value lower than s-HA, which may however be assigned to a difference in profile resulting from surface effects in the bone mineral nanocrystals with respect to s-HA powder. Eventually, the heated ns-HA powders exhibit a higher frequency and broader peak at ∼ 2.2 THz, confirming the presence of several phases in the samples identified in the diffraction patters. The widths of the lorentzian and gaussian contributions to the Voigt profile also follow interesting trends, as represented in Fig 5b. The overall (Voigt) width is found to decrease upon increasing temperature, which is associated with crystal growth. Besides, the width of the gaussian contribution decreases with the heating temperature, until a purely lorentzian profile for the s-HA sample. Such a profile evolution clearly indicates a heterogeneous (gaussian) broadening in the bone spectra with respect to synthetic crystals. This indicates that, even at high temperature, a non-negligible degree of disorder remains in bone mineral when compared to the idealized hydroxyapatite structure. Only the 900°C-aged sample shows significant deviations from those trends. At this stage, it is important to report on the aging of the sample and underline that further characterizations are needed to conclude on the nature of the process.

All those results are in perfect agreement with X-ray diffraction investigations widely spread in the literature, therefore proving THz-TDS to be a quantitative technique for phase and crystallinity investigations in bone tissues.

The investigation of commercial HA and of a series of synthetic calcium phosphate compounds related to apatite, with close chemical composition and structure, enables a deeper understanding and shows the broad utility of the technique. DFT normal mode calculation proves that all the investigated apatite-related compounds have normal mode in the THz range. Furthermore, other experimental investigations by inelastic neutron scattering in calcium-related compounds (calcite [52], CaCO_3_, calcium hydroxide [53], Ca(OH)_2_, and brushite [54, 55], CaHPO_4⋅_2H_2_O) show the presence of phonons of similar frequency. However, in the THz-TDS spectrum, only *β*-CPP and especially s-HA exhibit a narrow vibrational mode at the characteristic frequency of 2.1 THz. Similarly, a peak at ∼2 THz has been observed in cuttlefish bone [56] due to the mineral phase made of aragonite calcium carbonate (CaCO_3_). The normal mode displacements analysis based on our DFT calculations indicates an optical phonon, in good agreement with previous calculations [46] and phonon measurement [52]. These two studies indicate a very low group velocity *v*_*g*_ = *dω*/*dk* for this mode in the Brillouin zone center (Q lower than the inverse micron in our measurements), so that our measured phonon width, or inverse of the phonon lifetime, is poorly sensitive to the crystallite size. Both effects, size and disorder, are related to each other and are responsible for the peak broadening in ns-HA and cannot be disentangled in such measurements.

## Conclusion

In conclusion, we must understand that the presence of a 2.1 THz peak in the absorption spectra is not sufficient to prove the existence of HA crystals, since calcium—oxygen—phosphorous motions occur in a broad variety of crystal structures in a lattice mode. However, THz analysis can provide a simple and quantitative signature of a bone tissue that was heated at a temperature higher than ∼750°C, or that contains apatite crystallites larger than 100 nm. Thanks to it’s ease of implementation, THz-TDS could therefore be envisaged to screen large sample batches, either as a quality control during a graft manufacturing stage, or to evidence/confirm high temperature heating of bone fragments collected on archaeological sites. The technique could also be of great help in quantifying residuals of HA in biomaterials during their resorption without calcination, as for example in BCP^®^ implants.

## Supporting information

S1 Fig Refractive index from THz-TDS. Absorption coefficient and refractive index in bones at 1.5 THz as a function of heating temperature measured by THz-TDS.
(EPS)

S2 Fig FTIR spectroscopy. FTIR spectra of non stoichiometric hydroxyapatites (ns-HA). The samples were made of pellets of ns-HA powder as received and heated at 1000 °C in KBr.
(EPS)

S3 Fig SEM characterization. SEM images of s-HA, ns-HA as received and heated at 1000°C, CPP-*β*, *β*-TCP and TTCP. at three different scales: 100 *μ*m (left), 100 nm (center) and 20 nm (right).
(EPS)

S4 Fig EDX analysis. EDX characterization of three samples: s-HAP, ns-HA as received and ns-HA heated at 1000°C. The intensities are normalized on the Ca peak to highlight the calcium deficiency of the ns-HA with respect to s-HA. After heat treatment, the ratio C/P is changed but does not equal the one of s-HA because of the formation of *β*-TCP.
(EPS)
